# Prostacyclin Promotes Degenerative Pathology in a Model of Alzheimer’s Disease

**DOI:** 10.3389/fncel.2022.769347

**Published:** 2022-02-07

**Authors:** Tasha R. Womack, Craig T. Vollert, Odochi Ohia-Nwoko, Monika Schmitt, Saghi Montazari, Tina L. Beckett, David Mayerich, Michael Paul Murphy, Jason L. Eriksen

**Affiliations:** ^1^Department of Pharmacological and Pharmaceutical Sciences, University of Houston, Houston, TX, United States; ^2^Department of Molecular and Cellular Biochemistry, University of Kentucky, Lexington, KY, United States; ^3^Department of Electrical and Computer Engineering, University of Houston, Houston, TX, United States

**Keywords:** Alzheimer’s disease, prostanoid, amyloid-β, neuroinflammation, neurodegeneration

## Abstract

Alzheimer’s disease (AD) is a progressive neurodegenerative disorder that is the most common form of dementia in aged populations. A substantial amount of data demonstrates that chronic neuroinflammation can accelerate neurodegenerative pathologies. In AD, chronic neuroinflammation results in the upregulation of cyclooxygenase and increased production of prostaglandin H2, a precursor for many vasoactive prostanoids. While it is well-established that many prostaglandins can modulate the progression of neurodegenerative disorders, the role of prostacyclin (PGI2) in the brain is poorly understood. We have conducted studies to assess the effect of elevated prostacyclin biosynthesis in a mouse model of AD. Upregulated prostacyclin expression significantly worsened multiple measures associated with amyloid-β (Aβ) disease pathologies. Mice overexpressing both Aβ and PGI2 exhibited impaired learning and memory and increased anxiety-like behavior compared with non-transgenic and PGI2 control mice. PGI2 overexpression accelerated the development of Aβ accumulation in the brain and selectively increased the production of soluble Aβ_42_. PGI2 damaged the microvasculature through alterations in vascular length and branching; Aβ expression exacerbated these effects. Our findings demonstrate that chronic prostacyclin expression plays a novel and unexpected role that hastens the development of the AD phenotype.

## Introduction

Alzheimer’s disease is an incurable neurodegenerative disorder that is the most common cause of dementia. The late-onset form of AD is a slowly developing, progressive disorder, with age as the most significant single risk factor. Major pathological hallmarks of the disease include the accumulation of extracellular Aβ protein and intracellular tau protein, accompanied by prominent, widespread neuroinflammation (Itagaki et al., 1988; McGeer et al., 1988; Rogers et al., 1988; Eikelenboom et al., 1989). With disease progression, numerous neuroinflammatory molecules, including prostaglandins and cytokines, become dramatically upregulated within cerebrospinal fluid and throughout the brain parenchyma, and are associated with cognitive impairment (Casolini et al., 2002; Brüünsgaard and Pedersen, 2003; Starr et al., 2009; Cribbs et al., 2012). These findings suggest that persistent inflammation may be a driver of neurodegenerative disease (Holmes et al., 2009; Noble et al., 2010; Simen et al., 2011). The prostanoid family of lipid metabolites are the main mediators of acute inflammatory responses; however, their role in chronic inflammatory disease states remains controversial due to the dual role of many prostanoid receptors (Ricciotti and FitzGerald, 2011). For example, prostaglandin D2 (PGD_2_) bound to the PGD receptor (DP) stimulates mast cell secretion of type 2 cytokines in the lung leading to eosinophil infiltration and bronchoconstriction, clinically known as allergic asthma (Matsuoka et al., 2000). Conversely, in the CNS, DP1 receptor activation attenuated glutamate toxicity in organotypic hippocampal cultures indicating a neuroprotective role of prostanoid receptors (Liang et al., 2005b).

Prostanoids represent a broad class of arachidonic-acid-derived molecules, including thromboxane, prostaglandins, and prostacyclin, that can exhibit paracrine signaling activity. Prostanoid biosynthesis is carried out by cyclooxygenase (COX) enzymes. Typically, these prostanoids fill a variety of diverse physiological processes that are important for tissue homeostasis (Edelman et al., 2013; Liu et al., 2013; Vitale et al., 2013). Inflammatory events and neurotoxic insults can significantly upregulate prostanoid expression, a process that significantly contributes to the AD-associated neuroinflammatory response (Yermakova et al., 1999; Hoozemans et al., 2001; Choi and Bosetti, 2009; Coma et al., 2010). In Alzheimer’s disease, one of the crucial triggers associated with prostanoid up-regulation is the accumulation of Aβ protein, which both stimulates pro-inflammatory cytokine production and increases cellular phospholipase activity, the first step in prostanoid biosynthesis (Desbène et al., 2012; Shi et al., 2012; Sagy-Bross et al., 2013).

The liberation of fatty acid substrates, such as arachidonic acid, is the initiating step of the prostanoid signaling cascade. Free arachidonic acid undergoes conversion, in a two-step reaction, to prostaglandin H2 (PGH2) by COX enzymes. Two major COX enzymes exist, COX-1 and COX-2, which have distinct but overlapping tissue profiles and activities. COX-1 is expressed in the periphery and is a consistently active enzyme that is important for the maintenance of tissue homeostasis. In contrast, COX-2 represents an inducible form of the enzyme. In the brain, COX-2 expression usually is low but becomes dramatically upregulated in Alzheimer’s disease (Kitamura et al., 1999; Hoozemans et al., 2008). Prostaglandin E2 (PGE_2_), prostaglandin D2 (PGD_2_), prostacyclin (PGI_2_), prostaglandin F2α (PGF_2α_), and thromboxane A2 (TXA_2_) become upregulated with COX-2 expression; several of these prostanoids, such as PGE_2_ and PGD_2,_ can stimulate Aβ production and can play roles in other amyloid associated pathologies (Liang et al., 2005a; Hoshino et al., 2009; Keene et al., 2010; Zhen et al., 2012). However, the impact of increased PGI_2_ in AD is not well understood.

Prostacyclin is synthesized from arachidonic acid through a two-step reaction of the COX and PGI_2_ synthase (PGIS) enzymes. PGI_2_ acts *via* the G-protein coupled IP receptor to activate adenylyl cyclase and protein kinase A, thereby increasing intracellular cAMP to produce vasodilatory and anti-inflammatory effects (Cheng et al., 2002). While prostacyclin signaling is largely associated with peripheral vasoregulatory activity, multiple cell types in the brain express both PGIS and the IP receptor, including neurons, glia, endothelial cells, and smooth muscle cells (Oida et al., 1995; Siegle et al., 2000; Fang et al., 2006). These findings suggest that prostacyclin may act as a modulator of CNS activity.

Experimental applications of stable analogs of PGI_2_ within the brain have shown improvements in vascular functions and recovery from neuronal damage. For example, the application of iloprost, a stable analog of PGI_2,_ was able to significantly reduce infarct size after 6 h of middle cerebral artery occlusion in rabbits (Dogan et al., 1996). While the infusion of TEI-7165, another analog of PGI_2_, was able to rescue hippocampal neurons and improve response latencies in a step-down passive avoidance test in gerbils subjected to forebrain ischemia (Matsuda et al., 1997). Additionally, PGI_2_ appears to impact influence behavior and cognitive processing positively; CP-Tg mice contain a modified Cyclooxygenase-1 enzyme-linked to Prostacyclin-I synthase, which elevates levels of PGI_2_ production (Ruan et al., 2006, 2008; Ling et al., 2018) by 60% in the CNS (Vollert et al., 2014). In our previous work, we found that increased levels of PGI_2_ improved short-term memory as the CP-Tg mice learned significantly faster in training compared to controls in a contextual fear conditioning test (Vollert et al., 2014).

Several studies have suggested that PGI_2_ may have some impact on neurodegenerative disorders such as Alzheimer’s disease (Bush et al., 1990; Smith et al., 2004). He et al. (2017) was able to show that agonist-induced activation of the IP receptor stimulated production of soluble amyloid precursor protein α (sAβPPα), a neuroprotective isoform of the Aβ precursor protein (AβPP), in isolated human microvascular endothelial cells. Wang et al. (2016) demonstrated that injections of PGI_2_ into an AβPP/presenilin 1 transgenic mouse model increased Aβ levels and proposed this was due to upregulation of the anterior pharynx defective 1 homolog α (APH-1α) subunit of the AβPP cleaving protein, γ-secretase, via the PKA/CREB and JNK/c-Jun pathways. γ-secretases are responsible for cleavage of the AβPP to produce cytotoxic Aβ peptides, suggesting that PGI_2_ may regulate Aβ-associated pathology. These data suggest that increased PGI_2_ is likely to play a modulatory role in cognitive function associated with AβPP metabolism.

For this work, we evaluated the effect of PGI_2_-overexpression in a model of neurodegenerative disease. CP-Tg mice were crossed to APdE9 mice, a model of Alzheimer’s disease that develops prominent Aβ pathology and develops spatial memory impairments by 12 months of age (Lalonde et al., 2005). APdE9/CP-Tg mice, along with age-matched controls, were subjected to behavioral tests to assess possible changes in cognitive and anxiety-like behaviors. To investigate the impact of prostacyclin overexpression on the Aβ phenotype, we performed Aβ ELISAs on whole brain homogenates. We also investigated prostacyclin-mediated changes to the neurovasculature using immunohistochemical imaging.

## Materials and Methods

### Animal Model

All experiments were conducted following approved IACUC guidelines, using approved protocols, and mice were housed at the University of Houston Animal Care Facility. Mice were kept in group cages and exposed to a 12-h light/12-h dark cycle. To develop the APdE9/CP-Tg mouse model, CP-Tg mice, that express a hybrid enzyme complex linking COX-1 to PGIS by an amino acid linker of 10 residues (COX-1-10aa-PGIS; Ruan et al., 2006, 2008; Ling et al., 2018), were crossed to APdE9 mice, a bigenic model expressing the human APP Swedish mutation and the exon-9-deleted variant of presenilin-1 (dE9; Holcomb et al., 1998, 1999). PCR analysis was used to confirm the genotype. Heterozygous mice were used for all studies. Studies composed of a balanced mixture of male and female mice.

### Open Field Activity

The open-field test was used to analyze exploratory behavior within a 60 cm × 40 cm open chamber in normal lighting conditions. Each animal was placed in the center of the apparatus and was given 30 min to freely explore the arena. The movement was monitored by a computer-operated system (Optomax, Columbus Instruments, OH) that recorded the time each mouse spent moving, resting, or along the margin of the arena.

### Light Dark Exploration

As a measure of anxiety-like behavior, a single mouse is placed in an apparatus consisting of a light and dark compartment separated by a single opening and their movements are recorded (Vollert et al., 2014). Mice were subjected to light-dark exploration test to evaluate anxiety-like behavior at three and 6 months of age. The light-dark box consisted of a light compartment (27 cm × 27 cm × 27 cm) and a dark compartment (27 cm × 18 cm × 27 cm) separated by a partition with a single opening (7 cm × 7 cm) to allow passage between compartments as previously described More time spent in the dark compartment and fewer transitions is considered anxiogenic. Each trial lasted 5 min. The number of transitions were measured manually, with the observer blinded to the genotype.

### Elevated Plus-Maze

Anxiety-like behavior can be assessed by utilizing an elevated-plus maze where the mouse has the option to explore two open freely and lit arms, or two arms closed in with blinders. The plus-shaped apparatus is 40 cm above the ground, and mouse movements were recorded by an overhead camera. Time in the light and number of transitions between arms were manually recorded with the observer blinded to the genotype.

### Rotarod

An accelerating cylindrical drum Rotamex Rotarod machine (Columbus Instruments, Columbus, OH) was used to evaluate motor learning and coordination in 12-month-old animals. The rotarod consists of horizontal accelerating rods (4–40 rpm) and plastic partitions between each mouse. The mouse is then subjected to four trials a day for 2 days with 15-min intervals between each trial. A trial ended when the mouse fell off the rod, the time elapsed 300 s, or the mouse became inverted twice in the same trial without falling.

### Fear Conditioning

Testing for contextualized and cued fear conditioning was followed as previously described in mice at 6 months of age (Martinez et al., 2007; Elhardt et al., 2010). A standard fear conditioning chamber (13 × 10.5 × 13 cm, Med Associates) with 19 metal rods equally spaced on the floor was used to condition the mice. Training consisted of a 7-min session where a single mouse was subjected to a foot shock (2-s, 0.75 mA) paired with an auditory tone at 120, 240, and 360 s. To test for conditioning to contextual cues, the mouse was returned to the chamber 1 h and 24 h after the training session. The contextual tests were also 7-min sessions. However, the shock was not presented. For the 24-h cue test, mice were returned to the chamber after the surroundings and smell had been altered and during the 7-min session, a 3-min tone was presented from minute 3 to 6. Freezing behavior was measured using computer software (FreezeFrame, Med Associates/Actimetrics).

### Aβ ELISA

Mice were sacrificed after behavioral testing, and brains were collected. Briefly, hemibrains were homogenized in 1× PBS buffer (pH = 7.4, 1.0 ml/150 mg of tissue) using a PowerMax AHS 200 homogenizer and centrifuged at 14,000× *g* for 30 min at 4°C. The supernatant was collected, and the pellet re-extracted in RIPA buffer (50 mM Tris-HCl, 150 mM NaCl, 1% Triton X-100, 0.5% Deoxycholate, 0.1% SDS, 1× PIC, pH = 8.0) and the supernatant collected again. Sandwich ELISAs were performed for monomeric Aβ42 using the antibodies 2.1.3 (Aβ42 end specific, to capture) and Ab9 (human sequence Aβ1–16, to detect). Standards and samples were added to plates after coating the wells with 2.1.3 antibody in PBS and blocking with Synblock (Pierce). Detection antibodies were then applied and developed with TMB reagent (Kirkegaard and Perry Laboratories). The reaction was stopped using 6% *o*-phosphoric acid and read at 450 nm in a BioTek plate reader.

### Quantification of Aβ Pathology

4G8 antibody (1:500 dilution; Covance, cat no. SIG-39220) was used to detect Aβ pathology in 10 μm thick coronal brain slices. Slices were blocked for endogenous peroxidase using 3% hydrogen peroxide and blocked using 5% serum. Primary antibody was applied and incubated overnight at 4°C followed by a biotinylated secondary goat anti-mouse antibody (Jackson Immunoresearch) incubation for 20 min at room temperature and incubation with a Streptavidin/HRP label (Jackson Immunoresearch), followed by visualization with DAB.

Plaque expression in the entire cortex was quantified for each subject. Briefly, five brain sections, equally spaced through the cortex, from each subject were digitally captured and montaged at 10× using an Olympus DSU system using Neurolucia (Microbrightfield). The entire cortex was outlined, and Image J particle analysis with thresholding was used to quantify total Aβ burden, number, and average size for five sections. Studies were performed blinded for the genotype of each subject.

### Immunofluorescence Staining

The primary antibodies used were Collagen IV (1:400 dilution; Cosmo Bio, Catalog Number LSL-LB1403) and CD13 (1:100 dilution; R&D Systems, Catalog Number AF2335), NeuN (1:200 dilution; Millipore Sigma, Catalog Number MAB377 Clone A60), Iba 1 (1:200 dilution; FUJIFILM Wako Chemicals, Catalog Number 019-19741). Mice were sacrificed by decapitation, and brains were dissected. Brains were postfixed by immersion in Accustain (Sigma Aldrich). After fixation, brains were cryoprotected in 30% sucrose and cut into 60 μm thick sections with a cryostat. Free-floating coronal brain slices were subjected to a protein block at room temperature for 30 min. Slices were then incubated with primary antibodies at their respective concentrations overnight at 4°C, followed by consecutive incubations of biotinylated anti-rabbit secondary antibody (1:200; Jackson Immunoresearch) and biotinylated anti-goat secondary antibody (1:200; Jackson Immunoresearch) both for 1 h at room temperature. DyLight fluorophores (Jackson Immunoresearch) were added after each secondary incubation. Slices were then mounted onto glass slides under coverslips using Fluoro-Gel II mounting media with DAPI (Electron Microscopy Services).

### Confocal Microscopy Analysis

Quantification of vessel parameters and pericyte coverage was performed on 60 μm microtome brain slices. Five slices evenly spaced between plates 40 and 65 of the Franklin and Paxinos Mouse Brain Atlas (Franklin and Paxinos, 2019) were selected from each of five mice in each genotype for immunofluorescence staining. Blood vessels were visualized by collagen IV and pericytes by CD13 immunostaining. Three randomly selected areas of the cortex from each of the five slices were used for analysis. *Z*-stacks of 60 μm thickness were captured using a Confocal LS microscope (THOR Labs). Neurons were visualized by NeuN staining and three randomly selected areas of the cortex from each of three slices were used for analysis. Hyperactivated microglia were visualized by Iba 1 staining. Whole coronal brain slices were imaged and the entire cortex of three slices was used for analysis. NeuN and Iba 1 staining were captured at 20× as two-dimensional images using a Confocal LS microscope (Leica). Quantification of vessel parameters was performed using a blood vessel and network analysis plugin, Tube Analyst, in ImageJ (Tischer and Tosi, 2016). Pericyte coverage analysis was performed using the ImageJ JACoP plugin as previously described (Dunn et al., 2011). All *z*-stacks were processed with background subtraction and analyzed using automatic thresholding. Quantification of neuronal cell numbers was performed using ImageJ’s analyze particles feature after images were processed with background subtraction and a watershed mask. Hyperactivated microglia within the cortex were counted manually after blinding and randomization of the images. Iba 1-positive cells were only marked counted if a DAPI-positive nucleus was present.

### Statistical Analysis

Results for the contextualized and cued fear conditioning assays were analyzed using a repeated measures ANOVA; we analyzed all other data using factorial ANOVA followed by Fisher LSD *post hoc* comparisons using Statistica (Tibco Software). A *p* value less than 0.05 was considered significant. All values are represented as S.E.M.

## Results

### Impacts on Locomotor Activity and Anxiety-Like Behavior

**Figure 1:**
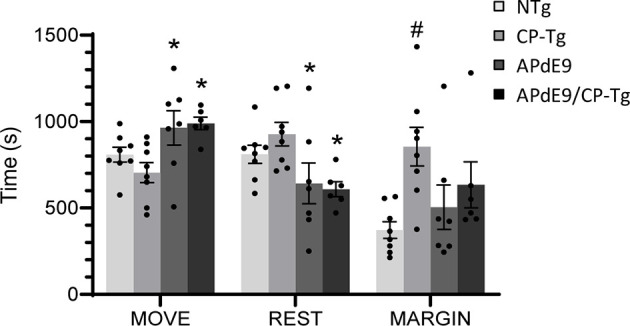
Effects of prostacyclin overexpression in the open-field test in APdE9 and control mice. Increases in ambulation and reduced resting times indicate a reduction in anxiety-like behavior (anxiolytic effect), with the opposite results indicating an increase in anxiety-like behavior (anxiogenic effect). Bars are means ± SEM, *n* = 7–8 mice per group. *p < 0.05 compared to CP-Tg control mice, ^#^*p* < 0.05 compared to NTg, APdE9, and APdE9/CP-Tg mice.

Analysis of open field data, a test for locomotor activity and anxiety-like behavior, revealed that ambulation, resting, and margin times differed among the mouse lines. CP-Tg mice exhibited significant anxiety-like behavior measured by greater times spent resting and in the margin with less time spent moving (Figure 1, *p* < 0.05). APdE9 lines showed significant increases in ambulation and reductions in resting times, indicating an anxiolytic-like effect when compared to the CP-Tg or non-transgenic (NTg) controls (Figure 1, *p* < 0.05). Prostacyclin overexpression in the APdE9/CP-Tg mice did not affect Aβ-mediated anxiety-like behavior as no differences were observed between the APdE9 mice and APdE9/CP-Tg mice (Figure 1).

**Figure 2:**
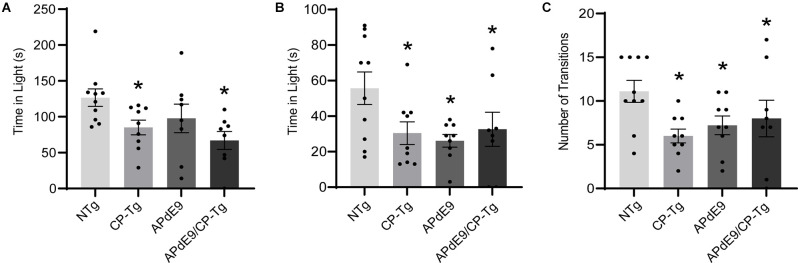
Prostacyclin overexpression increased anxiety-like behavior as measured by light-dark and elevated plus-maze tests. **(A)** CP-Tg and APdE9/CP-Tg mice spent significantly less time in the light during light-dark exploration. **(B)** In the elevated plus-maze, all transgenic mouse lines spent significantly less time in the lit arms and made fewer transitions between the open and closed arms **(C)** of the elevated plus-maze. Bars are means ± SEM, *n* = 8–10 mice per group. *p < 0.05 compared to NTg mice.

The light-dark exploration test and the elevated plus-maze were used to evaluate anxiety. In the light-dark exploration test, a decrease in exploratory behavior in the lighted area and a preference for the dark compartment was considered a measure of anxiety-like behavior. We found that prostacyclin overexpressing mice spent significantly less time in the light compartment than the NTg control mice (Figure 2A, *p* < 0.05). Anxiety-like behavior was also measured using an elevated plus-maze, with increased time in the lit open-arm of the plus-maze associated with anxiolytic behavior. Both the Aβ and prostacyclin expressing mice spent significantly less time in the open arms and made fewer transitions, indicative of anxiogenic behavior, compared to the non-transgenic control group (Figures 2B,C, *p* < 0.05).

### PGIS Overexpression Improves Motor Coordination

**Figure 3:**
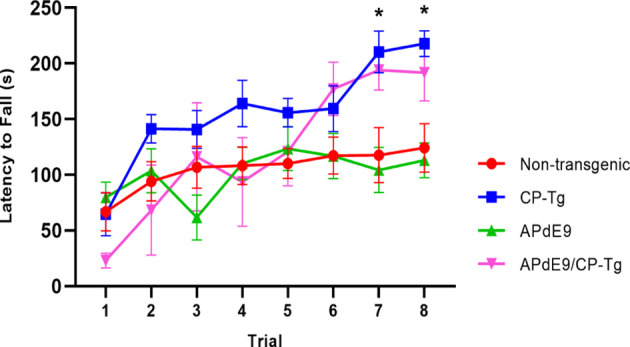
Prostacyclin overexpression enhances motor learning and coordination. In 14–17-month-old mice, both CP-Tg and APdE9/CP-Tg mice showed improved coordination on the rotarod, compared with NTg and APdE9 mice on trials 7 and 8. Bars are means ± SEM, *n* = 7–8 mice per group. *p < 0.05 compared to NTg and APdE9 mice.

Motor function and coordination were assessed using a motorized rotarod. CP-Tg and APdE9/CP-Tg mice had an increased latency to fall in trials 7 and 8 indicating PGIS overexpression increases coordination and balance in mice 14–17 months of age (Figure 3, *p* < 0.05).

### PGIS Overexpression Impairs Associative Learning

**Figure 4:**
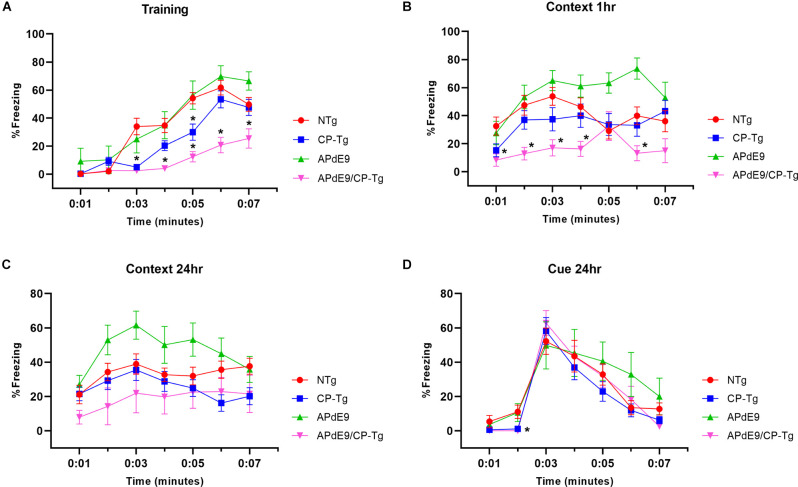
APdE9/CP-Tg mice exhibit impaired learning. **(A)** PGIS overexpression significantly decreased the percentage freezing time after the first and second shock (shock given at 2nd and 4th min) of the training trial (*p* < 0.05) while the combination of Aβ and prostacyclin further decreased freezing times during training. **(B)** APdE9 mice exhibited an enhanced short-term memory response while APdE9/CP-Tg mice exhibited a depressed short-term memory response. **(C)** CP-Tg and APdE9/CP-Tg mice exhibited worse performance in a contextual test of long-term memory compared to APdE9 mice. **(D)** CP-Tg and APdE9/CP-Tg mice showed an initial delay in freezing at the 2nd min; however, no significant differences were observed after application of the 3-min tone from minute 3 to 6 in a cued assessment of long-term memory. *N* = 7–14 mice per group. *p < 0.05 compared to NTg mice. PGIS, PGI2 synthase.

Context/cue fear conditioning tests were run to assess prostacyclin-mediated changes in associative learning of AD mice measured by percent freezing. During training, mice were conditioned with three shocks paired with a tone. The APdE9/CP-Tg mice displayed delayed learning to the aversive shocks when compared to the APdE9 mice as well as the CP-Tg and NTg controls as the magnitude of the freezing response was decreased in the training period (Figure 4A, *p* < 0.05). CP-Tg mice also demonstrated delayed learning compared to the APdE9, and NTg controls after the first and second shock but had an increased fear response from that of the APdE9/CP-Tg mice for the 3rd shock in the last 2 min (Figure 4A, *p* < 0.05). APdE9 mice maintained comparable learning to that of the NTg control mice with no differences seen in percentage freezing during the entire training trial (Figure 4A).

A contextual fear conditioning trial was performed 1 h after training. In the same environment but with no tone presented, APdE9/CP-Tg freezing responses were significantly reduced compared to that of NTg (Figure 4B, *p* < 0.05). The APdE9 mice showed a significant enhancement in freezing response during minutes 5 and 6 compared to the control (NTg and CP-Tg) mice (Figure 4B, *p* < 0.005).

To assess long-term memory consolidation, another contextual trial was performed 24 h after training. The APdE9 mice maintained a high percentage of freezing times like the results seen in the 1-h context trial (Figure 4C, *p* < 0.05). Percentage freezing times for the APdE9 mice were not significantly different from the NTg and CP-Tg controls. During a 24-h cued conditioning trial where a 3-min tone is presented in a different environment, all mice exhibited an increased freezing response to the tone with no significant differences by the end of the 7-min trial period. However, CP-Tg and APdE9/CP-Tg mice did exhibit significantly more activity measured by lower percentage freezing, compared with NTg and APdE9 mice, before the tone was presented (Figure 4D, *p* < 0.05).

### Prostacyclin Drives Aβ Production and Increases Amyloid Burden

**Figure 5:**
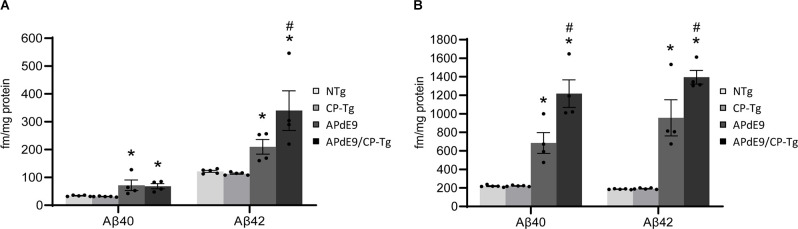
Prostacyclin overexpression increases Aβ production. PBS- **(A)** and RIPA-soluble **(B)** Aβ40 and Aβ42 levels in APdE9 mice with prostacyclin overexpression. Bars are means ± SEM, *n* = 10 mice per group. *p < 0.05 compared to control (non-transgenic and CP-Tg) mice. ^#^*p* < 0.05 compared to APdE9 mice. A two-way ANOVA with a Tukey’s HSD *post hoc* was used to determine significant differences.

An enzyme-linked immunosorbent assay of Aβ40 and Aβ42 was used to determine the impact of elevated PGI_2_ synthesis on Aβ production in the APdE9 mouse model. At 17–21 months of age, the APdE9 and APdE9/CP-Tg mice showed significant increases in both PBS-soluble levels of Aβ40 and −42 compared to the NTg or CP-Tg controls (Figure 5A, *p* < 0.05). When comparing the APdE9/CP-Tg mice to the APdE9 controls, the double transgenic line exhibited comparable levels of soluble Aβ40 and had a selective increase in soluble Aβ42 (Figure 5A, *p* < 0.05). Levels of insoluble Aβ40 and −42 in the brain were measured using a RIPA extraction. Again, the APdE9 and APdE9/CP-Tg mice had increased levels of both Aβ40 and −42 compared to the NTg or CP-Tg controls (Figure 5B, *p* < 0.0005), with the double transgenic line having an approximately 1.7-fold and 1.5-fold increases in insoluble Aβ40 and −42, respectively, compared to the APdE9 mice.

**Figure 6:**
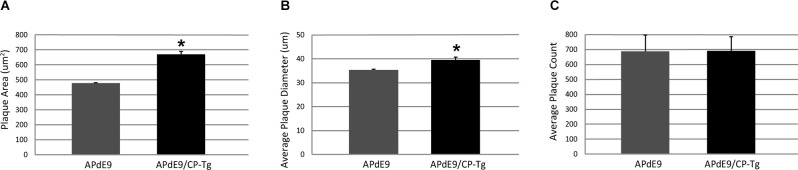
Prostacyclin overexpression increases plaque area and diameter in the brains of APdE9 mice. Measurements in five sections of the whole cortex determined **(A)** average plaque area, **(B)** average plaque diameter, and **(C)** cumulative plaque count per subject. Bars are means ± SEM, *n* = 10 mice per group. *p < 0.05 compared to APdE9 control mice. A one-way ANOVA was used to determine significant differences.

Increased production was reflected in higher Aβ burdens. Compared to the APdE9 mice, the APdE9/CP-Tg mice contained a significantly increased burden. The average plaque diameter in the APdE9/CP-Tg mice was significantly larger, with a mean 40% increase in plaque volume compared to APdE9 mice (Figure 6, *p* < 0.05). Although existing plaques were significantly larger, a notable finding from this study was that there was no significant difference in the number of plaques between different genetic lines.

### Loss of Pericyte Coverage in Prostacyclin-Overexpressing Lines

**Figure 7:**
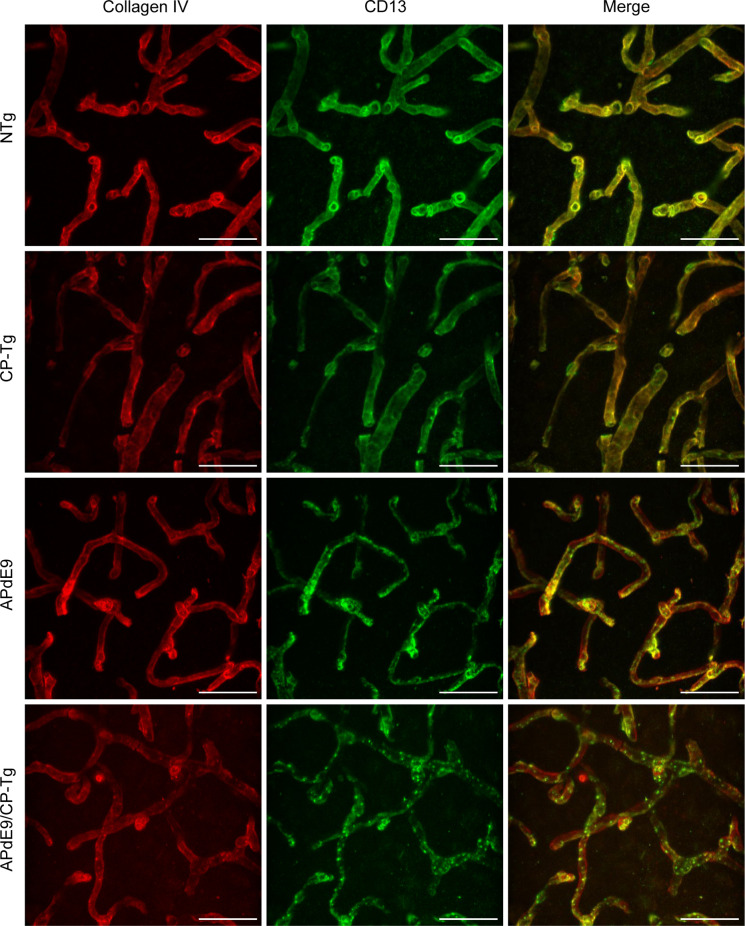
Loss of continuous pericyte coverage in APdE9/CP-Tg mice. 100× confocal image stacks of collagen IV-positive microvessels and CD13-positive pericytes from the cortex of 17 to 20-month-old NTg, CP-Tg, APdE9, and APdE9/CP-Tg mice. Scale bar = 30 μm.

A colocalization study was performed to assess the effect of prostacyclin overexpression on pericyte death in an AD mouse model. The colocalization of CD-13-positive pericytes with collagen IV-positive microvessels was expressed as Manders Colocalization Coefficients (MCC; M1: the fraction of CD-13 overlapping collagen IV, M2: the fraction of collagen IV overlapping CD-13; Dunn et al., 2011). In NTg mice, approximately 79% of CD-13 positive pericytes co-located with collagen IV-positive basement membrane, but in CP-Tg mice, this was significantly decreased (*F*_(3,95)_ = 13.41, *p* < 0.05) to nearly 73% (Table 1). Both Aβ-expressing models exhibited further reductions in pericyte coverage; APdE9 mice had 65% coverage, and APdE9/CP-Tg had 62% (Table 1, *p* < 0.05). Representative images of each genotype are presented in Figure 7.

**Table 1:** Effect of PGIS overexpression on colocalization of microvessels and pericytes.

	MCC M1		MCC M2		Pearson’s coefficient	
	Mean	SEM	Mean	SEM	Mean	SEM
NTg	0.808	0.012	0.788	0.020	0.859	0.013
CP-Tg	0.796	0.016	^#^0.728	0.022	0.774	0.019
APdE9	*0.852	0.011	*0.652	0.018	0.783	0.008
APdE9/CP-Tg	0.788	0.011	*0.624	0.023	0.704	0.016

Pearson’s and Manders Colocalization Coefficients (MCC) from dual immunofluorescent stains of collagen IV within the basement membrane of microvessels and CD-13 positive pericytes. M1 signifies the fraction of immunodetectable CD-13-positive pericytes overlapping Collagen IV-positive microvessels. M2 signifies the fraction of immunodetectable collagen IV-positive basement membrane overlapping CD-13-positive pericytes. *p < 0.05 compared to NTg and CP-Tg mice. ^#^*p* < 0.05 compared to NTg mice.

### APdE9/CP-Tg Mice Exhibit Severe Vascular Pathology

**Figure 8:**
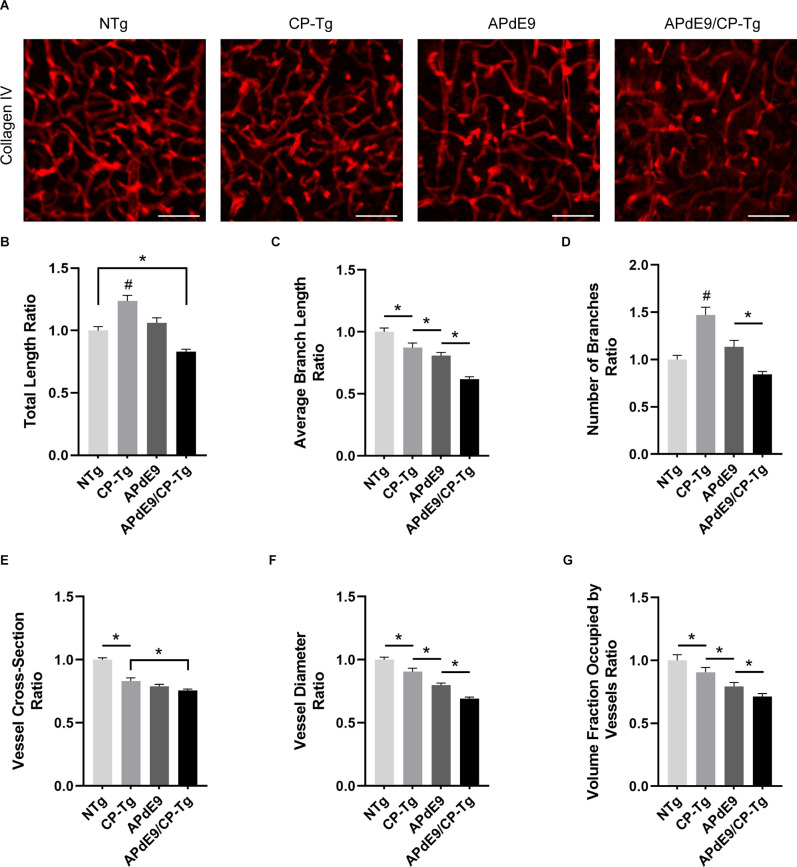
Cerebral vasculature structures are damaged by prostacyclin overexpression in APdE9/CP-Tg mice. Quantification of vascular structures, by collagen IV staining, in the cortex of 17–20-month-old NTg, CP-Tg, APdE9, and APdE9/CP-Tg mice. **(A)** 40 × 60 μm thick confocal image stacks of collagen IV-positive microvessels were used for vessel tracing analysis. Scale bar = 50 μm. **(B)** APdE9/CP-Tg mice had significantly fewer vessels compared to NTg or CP-Tg and APdE9 mice, while CP-Tg mice had significantly more. **(C)** APdE9/CP-Tg mice had significantly fewer number of vessel branches compared to NTg or CP-Tg and APdE9 mice, while CP-Tg mice had significantly more. **(D)** Branch length is significantly reduced in all models compared to NTg mice; CP-Tg mice show the least reduction in length while APdE9/CP-Tg mice show the greatest. **(E,F)** Cortical vessel cross-section and diameter were significantly smaller in all models compared to NTg mice with APdE9 mice exhibiting the greatest amount of smaller, constricted vessels. **(G)** Of the volume of vessels imaged for each genotype, NTg mice maintained the greatest fraction of vessels imaged while APdE9/CP-Tg mice contained the fewest. **(B–G)** All measures are a ratio of total vessel volume imaged by genotype and set to a normalized scale where control equals 1. *p < 0.05; ^#^*p* < 0.05 compared to NTg, APdE9, and APdE9/CP-Tg mice. Bars are means ± SEM, *n* = 5 mice per group.

To examine prostacyclin-mediated structural changes to the cerebral vasculature, we examined stains of the vessel basement membrane using confocal microscopy. Three-dimensional image stacks were analyzed using a vessel tracing software plugin in ImageJ. Vascular parameters, including total vessel length, branch number and length, vessel cross-section and diameter, and fractional vessel volume were quantified in the cortex of 17–20-month-old NTg, CP-Tg, APdE9, and APdE9/CP-Tg mice (Figure 8A). The vascular parameters are reported as a percent of the total vessel volume imaged. APdE9/CP-Tg mice had significantly shorter and fewer vessels compared to the other models (Figures 8B–D, *p* < 0.05). CP-Tg mice were found to have a significant increase in both total vessel length and the number of branches compared to NTg mice (Figures 8B,C, *p* < 0.05). APdE9/CP-Tg mice also presented with the smallest vessel cross-sections and diameters, APdE9 mice with the second smallest, and then CP-Tg mice when compared to NTg mice (Figures 8E,F, *p* < 0.05). The imaged volume fraction occupied by vessels was largest for NTg mice and smallest for APdE9/CP-Tg mice (Figure 8G, *p* < 0.05).

### Degenerative Pathology Associated With Prostacyclin Overexpression Is Not due to Cortical Neuronal Loss

**Figure 9:**
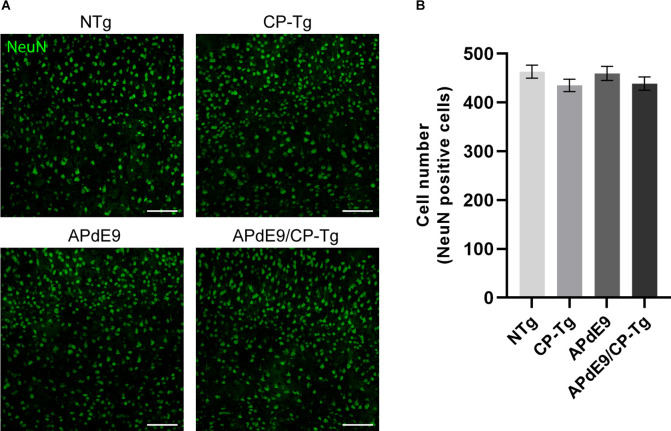
No changes in neuronal loss were detected in cortices of the transgenic mice when compared to the control mice. Quantification of neurons, by NeuN staining, in the cortex of 17–20-month-old NTg, CP-Tg, APdE9, and APdE9/CP-Tg mice. **(A)** 20× two-dimensional confocal images of NeuN-positive neurons were used for cell counting. Scale bar = 50 μm. **(B)** APdE9 mice and both prostacyclin overexpression models showed no loss of cortical neurons. Bars are means ± SEM, *n* = 4–5 mice per group.

Possible changes in the neuronal loss were assessed using neuronal cell staining and confocal microscopy. Two-dimensional images of cortical neurons were collected, and cell bodies were counted using the particle analyzer plugin in ImageJ. Cell counts are reported as the number of cells detectable in a 20× field of view (Figure 9A). No changes in neuronal cell numbers were detected in the cortex of any genotype when compared to the NTg control mice (Figure 9B).

### APdE9/CP-Tg Mice Have an Increased Neuroinflammatory Response

**Figure 10:**
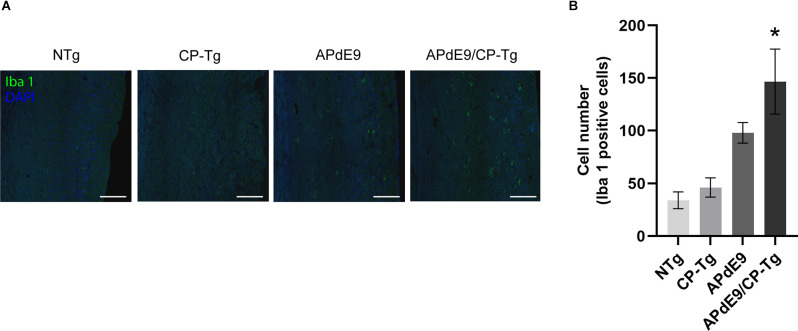
Increase of neuroinflammation in APdE9/CP-Tg mice. Quantification of activated microglia, measured by Iba 1 staining, in the cortex of 17–20-month-old NTg, CP-TG, APdE9, and APdE9/CP-Tg mice. **(A)** Representative two-dimensional images of Iba 1-positive microglia in whole coronal brain slices. Scale bar = 50 μm. **(B)** Prostacyclin overexpression in the APdE9 mouse model significantly increases neuroinflammation measured by the presence of hyperactivated microglia in brain tissues. *p < 0.05 compared to NTg mice. *n* = 4−5 mice per group.

Ionized calcium-binding adapter molecule 1 (Iba 1) is a factor present in activated microglia during an inflammatory response. Immunostaining with an Iba1 antibody in free-floating coronal brain slices showed significant increases of activated microglia in APdE9/CP-Tg mice in comparison with NTg mice (Figures 10A,B, *p* < 0.05). Manual quantification in ImageJ showed a 4-fold increase in Iba 1-positive cell counts in APdE9/CP-Tg mice compared to NTg mice. APdE9 mice exhibited an upward trend in activated microglia; however, this effect was not found to be significant (Figure 10B, *p* = 0.09).

## Discussion

In this study, we characterized the effect of upregulated PGI_2_ production on behavior, Aβ pathology, and vessel morphology in a mouse model of AD. Previous work has implicated that exogenous application of PGI_2_ recovers neurological activity in animal models of ischemia or stroke, improves cerebral blood flow, and prevents pericyte loss and vascular leakage after lipopolysaccharide-induced spinal cord injury in mice (Dogan et al., 1996; Matsuda et al., 1997; Muramatsu et al., 2015; Yang et al., 2017).

We first addressed the possible neuroprotective effects of PGI_2_ overexpression on cognitive and non-cognitive behaviors. In an open field assessment of locomotor activity, both Aβ-expressing lines (APdE9 and APdE9/CP-Tg), displayed hyperactivity when compared to the CP-Tg or NTg controls at 12 months of age. CP-Tg mice exhibited decreased activity and spent a larger amount of time along the margin of the field, similar to findings from our previous work (Vollert et al., 2014). PGI_2_ overexpression, alone and with the APdE9 phenotype, significantly improved coordination by the last two rotarod trials; we speculate that improvements in motor coordination may be due to increased IP receptor signal in regions involved in motor learning (i.e., striatopallidal and cerebellum) as elevated cAMP is associated with improved motor function (Augustin et al., 2014). Elevated plus-maze and light-dark tests were used to assess anxiety-like behavior. Both measures of anxiety-like behavior revealed increased anxiety in all transgenic models (CP-Tg, APdE9, and APdE9/CP-Tg) measured by time spent in the lit compartment and open arms. Previous reports of the APdE9 mouse model observed similar results as the APPswe/PS1dE9 mice displayed increased exploration in an open field and open arms of an elevated plus-maze (Lalonde et al., 2005). Notably, PGI_2_ overexpression did not affect anxiety-like behavior in the APdE9 model as the APdE9/CP-Tg mice performed similarly to APdE9 mice. However, PGI_2_ overexpression was enough to produce an anxiety phenotype.

Recent work has suggested that activation of the IP receptor, a cyclic adenosine monophosphate (cAMP)-dependent PKA pathway, may be sufficient to drive stress responses *in vivo* (Keil et al., 2016). Stimulation of this pathway leads to activation of cAMP-responsive elements binding proteins in the nucleus to synthesize new proteins that alter fear learning and memory formation (Keil et al., 2016). Increased expression of prostacyclin also appeared to exert detrimental effects on other cognitive tests. We observed impaired learning in APdE9/CP-Tg mice during an associative fear conditioning test. Mice were conditioned to a cue and then assessed for contextual and cued memory to the environment or a tone, respectively. During training, APdE9/CP-Tg mice exhibited a significantly reduced freezing response compared to controls indicating a delay in memory acquisition. Additionally, CP-Tg mice also showed delayed acquisition when compared to non-transgenic controls at the third and fifth minutes following the 2-min and 4-min tone-shock pairings. A hippocampal-dependent contextual test was performed 1 h and 24 h after training. APdE9/CP-Tg mice again showed reduced freezing response compared to all controls at 1 h, and 24 h were comparable with CP-Tg and non-transgenic controls.

Conversely, previous studies using an exogenous application of a prostacyclin analog, MRE-269, after stroke in aged rats, were able to recover long-term locomotor and somatosensory functions (Yang et al., 2017). Although IP receptor deletion in mice can be neuroprotective with acute insults such as ischemic damage (Wei et al., 2008), chronic PGI_2_ overexpression may exert adverse effects. Our work demonstrates that elevated levels of prostacyclin impair hippocampal memory function in the presence of Aβ insults. Fear behavior during a 24-h auditory cue test was comparable between all groups. As cued conditioning is hippocampal-independent as it requires the use of the amygdala, our results indicate the combination of Aβ, and prostacyclin expression has little to no effect on amygdala-mediated memory retention.

We also find that PGI_2_ overexpression significantly increases Aβ production in APdE9/CP-Tg mice. APdE9 mice begin to develop plaques around 6 months of age, with abundant plaques around 12 months of age, and show behavioral impairments in spatial learning by 12 months of age (Jankowsky et al., 2004). In this study, ELIZAs of whole-brain homogenates show a more than 1.5-fold increase in insoluble Aβ40 and Aβ42 in the double transgenic line compared to the APdE9 mice suggesting Aβ burden is directly affected by prostacyclin expression. Intriguingly, these increases are associated with a selective increase of Aβ42 in the soluble pool, suggesting that prostacyclin may selectively bias γ-secretase processing. Another interesting observation in this study was the finding that prostacyclin caused an average 40% increase in plaque diameter, but without a detectible increase in the overall number, perhaps suggesting that factors involved in Aβ metabolism may be modulated through the IP receptor. These findings are supported by recent work on prostacyclin signaling influencing APP processing mechanisms. Agonist activation of the IP receptor was found to increase the expression of the AβPP, resulting in the increased production of Aβ (He et al., 2017). Another report suggests that downstream activation of the PKA/CREB pathway after prostacyclin injections induces upregulation of γ-secretase cleaving enzymes and leads to an accumulation of Aβ c-terminal fragments in an APP/PS1 transgenic mouse model (Wang et al., 2016).

Substantial evidence has shown that disruptions to the neurovasculature are evident in humans and mouse models of Alzheimer’s disease (AD) that directly coincide with an earlier onset and accelerated progression of AD-related pathologies (Bell and Zlokovic, 2009; Zlokovic, 2011; Snyder et al., 2015). These disruptions include altered cerebrovascular functions such as reduced cerebral blood flow velocities and higher resistance indexes (Jin et al., 2017) that have been correlated with impaired cognition (Roher et al., 2012; Ezzati et al., 2017). Structural abnormalities include changes such as capillary atrophy, cell degeneration, loss of tight junction proteins within the blood-brain barrier, and basement membrane thickening due to cerebral amyloid angiopathy (CAA) in both mouse models and individuals with AD (Thal et al., 2008; Bell, 2012; Kelleher and Soiza, 2013; Sengillo et al., 2013; Lai et al., 2015). However, a complete description of how vascular pathology can affect AD progression, and the role of the inflammatory pathway in vascular and neuronal injuries, is still being investigated.

Using a volumetric analysis, we measured the impact of PGI_2_ overexpression on vascular structure within the brain, quantifying measures of vessels such as length, branching, diameter, and volume. Our results show that the combination of PGI_2_ overexpression with the Aβ phenotype was quite detrimental and worsened multiple measures of vascular structure. Compared with the control group, APdE9/CP-Tg mice had fewer vessels with shorter, smaller diameters. Compared to NTg mice, APdE9 mice had significantly smaller branch lengths, cross-sections, and diameters, as well as a reduction in total volume. Reductions in microvascular density and vessel constriction have been reported in individuals with AD and mouse models of AD. Other studies have suggested that Aβ insults can increase microvascular density in mice (Biron et al., 2013), and there is evidence of angiogenic vessels, accompanied by higher microvessel density, in the hippocampus of post-mortem tissues of individuals diagnosed with AD (Desai et al., 2009). Moreover, some studies have found no changes in vessel volume in both humans and APP/PS1 mice (Hooijmans et al., 2007; Hunter et al., 2012; Burke et al., 2014). In our studies, we also found an increase in total vessel length and degree of branching in the CP-Tg mice compared to their non-transgenic controls or both Aβ models. However, the CP-Tg mice exhibited significantly smaller diameters and cross-sections and had a lower volume than that of NTg mice, indicating that the overproduction of PGI_2_ is detrimental to the neurovasculature.

The regulatory function of the IP receptor in the vasculature of the brain is poorly understood. Consequently, alterations in vasculature led us to examine perivascular cells or pericytes of the BBB. These cells maintain the integrity of the cerebral vasculature by promoting tight junction protein expression, facilitate cell-to-cell alignment, and are integral to preventing leakage of neurotoxic macromolecules that lead to neuronal damage (Bell et al., 2010). Application of PGI_2_ after lipopolysaccharide-induced vascular damage in a mouse model of spinal cord injury was able to rescue pericyte loss and concurrent leakage of blood components into the central nervous system (Muramatsu et al., 2015). Previous work has reported that accumulation of Aβ in the neurovasculature of AD individuals was correlated with reduced coverage of pericytes on capillaries in AD brains compared to controls (Sengillo et al., 2013). Furthermore, studies of pericyte-deficient, APP^*sw/0*^ (Swedish mutation) mice showed that they had accelerated levels of Aβ deposition, increased tau pathology, and increased neuronal degeneration by 9 months of age when compared to AD control mice (Sagare et al., 2013). In our studies, we evaluated the percentage of CD-13-positive pericytes that co-located with the vessels’ basement membrane as a measure of coverage. CP-Tg mice exhibited a significant reduction in pericyte coverage compared to non-transgenic controls, demonstrating that PGI_2_ plays a modulatory role in pericyte function. We identified significant reductions in pericyte coverage of APdE9 and APdE9/CP-Tg mice compared to NTg and CP-Tg mice. However, no difference was observed between APdE9 and APdE9/CP-Tg mice, indicating that Aβ-dependent reductions in pericyte coverage are unaffected by prostacyclin overproduction.

We conclude that PGI_2_ expression worsened multiple measures associated with degenerative pathology. We observed that PGI_2_ overexpression influences anxiety, possibly due to increases in cAMP activity that has been reported to alter gene expression of proteins involved with the fear response. APdE9 and APdE9/CP-Tg mice exhibited the same level of anxiety as the CP-Tg mice in the light-dark and elevated-plus maze behavioral tests suggesting PGI_2_ does not increase the anxiety effect observed in the AD model. In a fear conditioning test, the APdE9/CP-Tg mice exhibited significantly worse associative memory acquisition and consolidation than APdE9 controls. Although earlier reports using PGI_2_ analogs suggest activation of the IP receptor could be neuroprotective, CP-Tg mice presented with memory deficits when compared to non-transgenic controls; degenerative pathology was not associated with neuronal loss. In our studies, increased production of PGI_2_ accelerated amyloidogenesis by more than 50%, significantly increasing the production of soluble and insoluble Aβ peptides. Overexpression of PGI_2_ severely impacted multiple measures of vascular health, a process that was exacerbated with Aβ pathology. Given that prostacyclin is largely protective when expressed in peripheral tissues, our observations suggest that IP signaling is fundamentally different in the cerebrovasculature. Neuroinflammation was also synergistically elevated in the double transgenic model that was not seen with prostacyclin or amyloid overexpression alone. This suggests an immunomodulatory role for prostacyclin signaling in AD. Future studies should explore the mechanism of PGI_2_ signaling through its respective receptor within specific cell types of the CNS to better understand the role PGI_2_ plays in influencing the progression of neurodegenerative disease.

## Data Availability Statement

The raw data supporting the conclusions of this article will be made available by the authors, without undue reservation.

## Ethics Statement

The animal study was reviewed and approved by Institutional Animal Care and Use Committee, University of Houston.

## Author Contributions

TW, OO-N, MM, DM, and JE designed research. TW, OO-N, CV, SM, MS, and TB performed research. TW, CV, OO-N, TB, and JE analyzed data. TW and JE wrote the article. All authors contributed to the article and approved the submitted version.

## Conflict of Interest

The authors declare that the research was conducted in the absence of any commercial or financial relationships that could be construed as a potential conflict of interest.

## Publisher’s Note

All claims expressed in this article are solely those of the authors and do not necessarily represent those of their affiliated organizations, or those of the publisher, the editors and the reviewers. Any product that may be evaluated in this article, or claim that may be made by its manufacturer, is not guaranteed or endorsed by the publisher.

## References

[B1] Augustin S. M. Beeler J. A. McGehee D. S. Zhuang X. (2014). Cyclic AMP and afferent activity govern bidirectional synaptic plasticity in striatopallidal neurons. J. Neurosci. 34, 6692–6699. 10.1523/JNEUROSCI.3906-13.2014 24806695 PMC4012319

[B3] Bell R. D. (2012). The imbalance of vascular molecules in Alzheimer’s disease. J. Alzheimers Dis. 32, 699–709. 10.3233/JAD-2012-121060 22850315

[B4] Bell R. D. Winkler E. A. Sagare A. P. Singh I. LaRue B. Deane R.. (2010). Pericytes control key neurovascular functions and neuronal phenotype in the adult brain and during brain aging. Neuron 68, 409–427. 10.1016/j.neuron.2010.09.043 21040844 PMC3056408

[B2] Bell R. D. Zlokovic B. V. (2009). Neurovascular mechanisms and blood-brain barrier disorder in Alzheimer’s disease. Acta Neuropathol. 118, 103–113. 10.1007/s00401-009-0522-3 19319544 PMC2853006

[B5] Biron K. E. Dickstein D. L. Gopaul R. Fenninger F. Jefferies W. A. (2013). Cessation of neoangiogenesis in Alzheimer’s disease follows amyloid-beta immunization. Sci. Rep. 3:1354. 10.1038/srep01354 23446889 PMC3584312

[B6] Brüünsgaard H. Pedersen B. K. (2003). Age-related inflammatory cytokines and disease. Immunol. Allergy Clin. North Am. 23, 15–39. 10.1016/s0889-8561(02)00056-5 12645876

[B7] Burke M. J. C. Nelson L. Slade J. Y. Oakley A. E. Khundakar A. A. Kalaria R. N. (2014). Morphometry of the hippocampal microvasculature in post-stroke and age-related dementias. Neuropathol. Appl. Neurobiol. 40, 284–295. 10.1111/nan.12085 24003901 PMC4282329

[B8] Bush A. I. Martins R. N. Rumble B. Moir R. Fuller S. Milward E.. (1990). The amyloid precursor protein of Alzheimer’s disease is released by human platelets. J. Biol. Chem. 265, 15977–15983. 2118534

[B9] Casolini P. Catalani A. Zuena A. R. Angelucci L. (2002). Inhibition of COX-2 reduces the age-dependent increase of hippocampal inflammatory markers, corticosterone secretion and behavioral impairments in the rat. J. Neurosci. Res. 68, 337–343. 10.1002/jnr.10192 12111864

[B10] Cheng Y. Austin S. C. Rocca B. Koller B. H. Coffman T. M. Grosser T.. (2002). Role of prostacyclin in the cardiovascular response to thromboxane A2. Science 296, 539–541. 10.1126/science.1068711 11964481

[B11] Choi S.-H. Bosetti F. (2009). Cyclooxygenase-1 null mice show reduced neuroinflammation in response to β-Amyloid. Aging (Albany NY) 1, 234–244. 10.18632/aging.100021 20157512 PMC2806008

[B12] Coma M. Serenó L. Da Rocha-Souto B. Scotton T. C. España J. Sánchez M. B.. (2010). Triflusal reduces dense-core plaque load, associated axonal alterations and inflammatory changes and rescues cognition in a transgenic mouse model of Alzheimer’s disease. Neurobiol. Dis. 38, 482–491. 10.1016/j.nbd.2010.01.019 20149872 PMC3707138

[B13] Cribbs D. H. Berchtold N. C. Perreau V. Coleman P. D. Rogers J. Tenner A. J.. (2012). Extensive innate immune gene activation accompanies brain aging, increasing vulnerability to cognitive decline and neurodegeneration: a microarray study. J. Neuroinflammation 9:179. 10.1186/1742-2094-9-179 22824372 PMC3419089

[B14] Desai B. S. Schneider J. A. Li J.-L. Carvey P. M. Hendey B. (2009). Evidence of angiogenic vessels in Alzheimer’s disease. J. Neural Transm. (Vienna) 116, 587–597. 10.1007/s00702-009-0226-9 19370387 PMC2753398

[B15] Desbène C. Malaplate-Armand C. Youssef I. Garcia P. Stenger C. Sauvée M.. (2012). Critical role of CPLA2 in Aβ oligomer-induced neurodegeneration and memory deficit. Neurobiol. Aging 33, 1123.e17–1123.e29. 10.1016/j.neurobiolaging.2011.11.008 22188721

[B16] Dogan A. Temiz C. Türker R. K. Egemen N. Başkaya M. K. (1996). Effect of the prostacyclin analogue, iloprost, on infarct size after permanent focal cerebral ischemia. Gen. Pharmacol. 27, 1163–1166. 10.1016/s0306-3623(96)00051-1 8981062

[B17] Dunn K. W. Kamocka M. M. McDonald J. H. (2011). A practical guide to evaluating colocalization in biological microscopy. Am. J. Physiol. Cell Physiol. 300, C723–C742. 10.1152/ajpcell.00462.2010 21209361 PMC3074624

[B18] Edelman A. B. Jensen J. T. Doom C. Hennebold J. D. (2013). Impact of the prostaglandin synthase-2 inhibitor celecoxib on ovulation and luteal events in women. Contraception 87, 352–357. 10.1016/j.contraception.2012.07.004 22902348 PMC4040982

[B19] Eikelenboom P. Hack C. E. Rozemuller J. M. Stam F. C. (1989). Complement activation in Amyloid plaques in Alzheimer’s dementia. Virchows Arch. B. Cell Pathol. 56, 259–262. 10.1007/BF02890024 2565620

[B20] Elhardt M. Martinez L. Tejada-Simon M. V. (2010). Neurochemical, behavioral and architectural changes after chronic inactivation of NMDA receptors in mice. Neurosci. Lett. 468, 166–171. 10.1016/j.neulet.2009.10.091 19895868 PMC2787724

[B21] Ezzati A. Wang C. Lipton R. B. Altschul D. Katz M. J. Dickson D. W.. (2017). Association between vascular pathology and rate of cognitive decline independent of Alzheimer’s disease pathology. J. Am. Geriatr. Soc. 65, 1836–1841. 10.1111/jgs.14903 28407205 PMC5555777

[B23] Fang Y.-C. Wu J.-S. Chen J.-J. Cheung W.-M. Tseng P.-H. Tarn K.-B.. (2006). Induction of prostacyclin/PGI2 synthase expression after cerebral ischemia-reperfusion. J. Cereb. Blood Flow Metab. 26, 491–501. 10.1038/sj.jcbfm.9600205 16094316

[B39] Franklin K. Paxinos G. (2019). Paxinos and Franklin’s the Mouse Brain in Stereotaxic Coordinates, Compact - 5th Edition. Amsterdam: Academic Press. Available online at: https://www.elsevier.com/books/paxinos-and-franklins-the-mouse-brain-in-stereotaxic-coordinates-compact/franklin/978-0-12-816159-3. Accessed November 22, 2020.

[B24] He T. Santhanam A. V. R. Lu T. d’ Uscio L. V. Katusic Z. S. (2017). Role of prostacyclin signaling in endothelial production of soluble Amyloid precursor protein-α in cerebral microvessels. J. Cereb. Blood Flow Metab. 37, 106–122. 10.1177/0271678X15618977 26661245 PMC5363732

[B25] Holcomb L. A. Gordon M. N. Jantzen P. Hsiao K. Duff K. Morgan D. (1999). Behavioral changes in transgenic mice expressing both Amyloid precursor protein and presenilin-1 mutations: lack of association with amyloid deposits. Behav. Genet. 29, 177–185. 10.1023/a:1021691918517 10547924

[B26] Holcomb L. Gordon M. N. McGowan E. Yu X. Benkovic S. Jantzen P.. (1998). Accelerated Alzheimer-type phenotype in transgenic mice carrying both mutant amyloid precursor protein and presenilin 1 transgenes. Nat. Med. 4, 97–100. 10.1038/nm0198-097 9427614

[B27] Holmes C. Cunningham C. Zotova E. Woolford J. Dean C. Kerr S.. (2009). Systemic inflammation and disease progression in Alzheimer disease. Neurology 73, 768–774. 10.1212/WNL.0b013e3181b6bb95 19738171 PMC2848584

[B28] Hooijmans C. R. Graven C. Dederen P. J. Tanila H. Groen T. v. Kiliaan A. J. (2007). Amyloid beta deposition is related to decreased glucose transporter-1 levels and hippocampal atrophy in brains of aged APP/PS1 mice. Brain Res. 1181, 93–103. 10.1016/j.brainres.2007.08.063 17916337

[B29] Hoozemans J. J. M. Rozemuller J. M. Haastert E. S. v. Veerhuis R. Eikelenboom P. (2008). Cyclooxygenase-1 and -2 in the different stages of Alzheimer’s disease pathology. Curr. Pharm. Des. 14, 1419–1427. 10.2174/138161208784480171 18537664

[B30] Hoozemans J. J. Rozemuller A. J. Janssen I. De Groot C. J. Veerhuis R. Eikelenboom P. (2001). Cyclooxygenase expression in microglia and neurons in Alzheimer’s disease and control brain. Acta Neuropathol. 101, 2–8. 10.1007/s004010000251 11194936

[B31] Hoshino T. Namba T. Takehara M. Nakaya T. Sugimoto Y. Araki W.. (2009). Prostaglandin E2 stimulates the production of Amyloid-beta peptides through internalization of the EP4 receptor. J. Biol. Chem. 284, 18493–18502. 10.1074/jbc.M109.003269 19407341 PMC2709369

[B32] Hunter J. M. Kwan J. Malek-Ahmadi M. Maarouf C. L. Kokjohn T. A. Belden C.. (2012). Morphological and pathological evolution of the brain microcirculation in aging and Alzheimer’s disease. PLoS One 7:e36893. 10.1371/journal.pone.0036893 22615835 PMC3353981

[B33] Itagaki S. McGeer P. L. Akiyama H. (1988). Presence of T-cytotoxic suppressor and leucocyte common antigen positive cells in Alzheimer’s disease brain tissue. Neurosci. Lett. 91, 259–264. 10.1016/0304-3940(88)90690-8 2972943

[B34] Jankowsky J. L. Fadale D. J. Anderson J. Xu G. M. Gonzales V. Jenkins N. A.. (2004). Mutant presenilins specifically elevate the levels of the 42 residue beta-amyloid peptide *in vivo*: evidence for augmentation of a 42-Specific gamma secretase. Hum. Mol. Genet. 13, 159–170. 10.1093/hmg/ddh019 14645205

[B36] Jin W.-S. Bu X.-L. Wang Y.-R. Li L. Li W.-W. Liu Y.-H.. (2017). Reduced cardiovascular functions in patients with Alzheimer’s disease. J. Alzheimers Dis. 58, 919–925. 10.3233/JAD-170088 28505975

[B37] Keene C. D. Chang R. C. Lopez-Yglesias A. H. Shalloway B. R. Sokal I. Li X.. (2010). Suppressed accumulation of cerebral amyloid β peptides in aged transgenic Alzheimer’s disease mice by transplantation with wild-type or prostaglandin E2 receptor subtype 2-Null bone marrow. Am. J. Pathol. 177, 346–354. 10.2353/ajpath.2010.090840 20522650 PMC2893677

[B38] Keil M. F. Briassoulis G. Stratakis C. A. (2016). The role of protein kinase A in anxiety behaviors. Neuroendocrinology 103, 625–639. 10.1159/000444880 26939049

[B40] Kelleher R. J. Soiza R. L. (2013). Evidence of endothelial dysfunction in the development of Alzheimer’s disease: is Alzheimer’s a vascular disorder? Am. J. Cardiovasc. Dis. 3, 197–226. 24224133 PMC3819581

[B41] Kitamura Y. Shimohama S. Koike H. Kakimura J. i Matsuoka Y. Nomura Y.. (1999). Increased expression of cyclooxygenases and peroxisome proliferator-activated receptor-gamma in Alzheimer’s disease brains. Biochem. Biophys. Res. Commun. 254, 582–586. 10.1006/bbrc.1998.9981 9920782

[B42] Lai A. Y. Dorr A. Thomason L. A. M. Koletar M. M. Sled J. G. Stefanovic B.. (2015). Venular degeneration leads to vascular dysfunction in a transgenic model of Alzheimer’s disease. Brain 138, 1046–1058. 10.1093/brain/awv023 25688079

[B43] Lalonde R. Kim H. D. Maxwell J. A. Fukuchi K. (2005). Exploratory activity and spatial learning in 12-month-old APP(695)SWE/Co+PS1/DeltaE9 mice with amyloid plaques. Neurosci. Lett. 390, 87–92. 10.1016/j.neulet.2005.08.028 16169151

[B44] Liang X. Wang Q. Hand T. Wu L. Breyer R. M. Montine T. J.. (2005a). Deletion of the prostaglandin E2 EP2 receptor reduces oxidative damage and amyloid burden in a model of Alzheimer’s disease. J. Neurosci. 25, 10180–10187. 10.1523/JNEUROSCI.3591-05.2005 16267225 PMC6725803

[B45] Liang X. Wu L. Hand T. Andreasson K. (2005b). Prostaglandin D2 mediates neuronal protection *via* the DP1 receptor. J. Neurochem. 92, 477–486. 10.1111/j.1471-4159.2004.02870.x 15659218

[B46] Ling Q.-L. Mohite A. J. Murdoch E. Akasaka H. Li Q.-Y. So S.-P.. (2018). Creating a mouse model resistant to induced ischemic stroke and cardiovascular damage. Sci. Rep. 8:1653. 10.1038/s41598-018-19661-y 29374184 PMC5786049

[B47] Liu B. Zhang Y. Zhu N. Li H. Luo W. Zhou Y. (2013). A vasoconstrictor role for cyclooxygenase-1-mediated prostacyclin synthesis in mouse renal arteries. Am. J. Physiol. Renal Physiol. 305, F1315–1322. 10.1152/ajprenal.00332.2013 23986518

[B48] Martinez L. A. Klann E. Tejada-Simon M. V. (2007). Translocation and activation of Rac in the hippocampus during associative contextual fear learning. Neurobiol. Learn. Mem. 88, 104–113. 10.1016/j.nlm.2007.01.008 17363298

[B50] Matsuoka T. Hirata M. Tanaka H. Takahashi Y. Murata T. Kabashima K.. (2000). Prostaglandin D2 as a mediator of allergic asthma. Science 287, 2013–2017. 10.1126/science.287.5460.2013 10720327

[B49] Matsuda S. Wen T.-C. Karasawa Y. Araki H. Otsuka H. Ishihara K.. (1997). Protective effect of a prostaglandin I2 analog, TEI-7165, on ischemic neuronal damage in gerbils. Brain Res. 769, 321–328. 10.1016/s0006-8993(97)00724-5 9374201

[B51] McGeer P. L. Itagaki S. Boyes B. E. McGeer E. G. (1988). Reactive microglia are positive for HLA-DR in the substantia nigra of Parkinson’s and Alzheimer’s disease brains. Neurology 38, 1285–1291. 10.1212/wnl.38.8.1285 3399080

[B52] Muramatsu R. Kuroda M. Matoba K. Lin H. Takahashi C. Koyama Y.. (2015). Prostacyclin prevents pericyte loss and demyelination induced by lysophosphatidylcholine in the central nervous system. J. Biol. Chem. 290, 11515–11525. 10.1074/jbc.M114.587253 25795781 PMC4416855

[B53] Noble J. M. Manly J. J. Schupf N. Tang M. X. Mayeux R. Luchsinger J. A. (2010). Association of C-reactive protein to cognitive impairment. Arch. Neurol. 67, 87–92. 10.1001/archneurol.2009.308 20065134 PMC4426905

[B54] Oida H. Namba T. Sugimoto Y. Ushikubi F. Ohishi H. Ichikawa A.. (1995). *In situ* hybridization studies of prostacyclin receptor MRNA expression in various mouse organs. Br. J. Pharmacol. 116, 2828–2837. 10.1111/j.1476-5381.1995.tb15933.x 8680713 PMC1909220

[B55] Ricciotti E. FitzGerald G. A. (2011). Prostaglandins and inflammation. Arterioscler. Thromb. Vasc. Biol. 31, 986–1000. 10.1161/ATVBAHA.110.207449 21508345 PMC3081099

[B56] Rogers J. Luber-Narod J. Styren S. D. Civin W. H. (1988). Expression of immune system-associated antigens by cells of the human central nervous system: relationship to the pathology of Alzheimer’s disease. Neurobiol. Aging 9, 339–349. 10.1016/s0197-4580(88)80079-4 3263583

[B57] Roher A. E. Debbins J. P. Malek-Ahmadi M. Chen K. Pipe J. G. Maze S.. (2012). Cerebral blood flow in Alzheimer’s disease. Vasc. Health Risk Manag. 8, 599–611. 10.2147/VHRM.S34874 23109807 PMC3481957

[B58] Ruan K.-H. Deng H. So S.-P. (2006). Engineering of a protein with cyclooxygenase and prostacyclin synthase activities that converts arachidonic acid to prostacyclin. Biochemistry 45, 14003–14011. 10.1021/bi0614277 17115695

[B59] Ruan K.-H. Jiaxin W. Cervantes V. (2008). Characterization of the substrate mimic bound to engineered prostacyclin synthase in solution using high-resolution NMR spectroscopy and mutagenesis: implication of the molecular mechanism in biosynthesis of prostacyclin. Biochemistry 47, 680–688. 10.1021/bi701671q 18081314

[B61] Sagare A. P. Bell R. D. Zhao Z. Ma Q. Winkler E. A. Ramanathan A.. (2013). Pericyte loss influences Alzheimer-like neurodegeneration in mice. Nat. Commun. 4:2932. 10.1038/ncomms3932 24336108 PMC3945879

[B62] Sagy-Bross C. Hadad N. Levy R. (2013). Cytosolic phospholipase A2α upregulation mediates apoptotic neuronal death induced by aggregated amyloid-β peptide1–42. Neurochem. Int. 63, 541–550. 10.1016/j.neuint.2013.09.007 24044897

[B63] Sengillo J. D. Winkler E. A. Walker C. T. Sullivan J. S. Johnson M. Zlokovic B. V. (2013). Deficiency in mural vascular cells coincides with blood-brain barrier disruption in Alzheimer’s disease. Brain Pathol. 23, 303–310. 10.1111/bpa.12004 23126372 PMC3628957

[B64] Shi J. Wang Q. Johansson J. U. Liang X. Woodling N. S. Priyam P.. (2012). Inflammatory prostaglandin E2 signaling in a mouse model of Alzheimer disease. Ann. Neurol. 72, 788–798. 10.1002/ana.23677 22915243 PMC3509238

[B65] Siegle I. Klein T. Zou M. H. Fritz P. Kömhoff M. (2000). Distribution and cellular localization of prostacyclin synthase in human brain. J. Histochem. Cytochem. 48, 631–641. 10.1177/002215540004800507 10769047

[B66] Simen A. A. Bordner K. A. Martin M. P. Moy L. A. Barry L. C. (2011). Cognitive dysfunction with aging and the role of inflammation. Ther. Adv. Chronic Dis. 2, 175–195. 10.1177/2040622311399145 23251749 PMC3513880

[B67] Smith C. C. T. Stanyer L. Betteridge D. J. (2004). Soluble β-amyloid (Aβ) 40 causes attenuation or potentiation of noradrenaline-induced vasoconstriction in rats depending upon the concentration employed. Neurosci. Lett. 367, 129–132. 10.1016/j.neulet.2004.05.094 15308313

[B68] Snyder H. M. Corriveau R. A. Craft S. Faber J. E. Greenberg S. M. Knopman D.. (2015). Vascular contributions to cognitive impairment and dementia including Alzheimer’s disease. Alzheimers Dement. 11, 710–717. 10.1016/j.jalz.2014.10.008 25510382 PMC4731036

[B69] Starr M. E. Evers B. M. Saito H. (2009). Age-associated increase in cytokine production during systemic inflammation: adipose tissue as a major source of IL-6. J. Gerontol. A. Biol. Sci. Med. Sci. 64, 723–730. 10.1093/gerona/glp046 19377014 PMC2844135

[B70] Thal D. R. Griffin W. S. T. Vos R. A. I. de. Ghebremedhin E. (2008). Cerebral amyloid angiopathy and its relationship to Alzheimer’s disease. Acta Neuropathol. 115, 599–609. 10.1007/s00401-008-0366-2 18369648

[B60] Tischer C. Tosi S. (2016). “Tumor blood vessels: 3d tubular network analysis,” in Bioimage Data Analysis ed Miura K. (Weinheim, Germany: Wiley-VCH), 219–236.

[B71] Vitale P. Tacconelli S. Perrone M. G. Malerba P. Simone L. Scilimati A.. (2013). Synthesis, pharmacological characterization and docking analysis of a novel family of diarylisoxazoles as highly selective cyclooxygenase-1 (COX-1) inhibitors. J. Med. Chem. 56, 4277–4299. 10.1021/jm301905a 23651359

[B72] Vollert C. Ohia O. Akasaka H. Berridge C. Ruan K.-H. Eriksen J. L. (2014). Elevated prostacyclin biosynthesis in mice impacts memory and anxiety-like behavior. Behav. Brain Res. 258, 138–144. 10.1016/j.bbr.2013.10.012 24140503 PMC3849419

[B73] Wang P. Guan P.-P. Guo J.-W. Cao L.-L. Xu G.-B. Yu X.. (2016). Prostaglandin I2 upregulates the expression of anterior pharynx-defective-1α and anterior pharynx-defective-1β in amyloid precursor protein/presenilin 1 transgenic mice. Aging Cell 15, 861–871. 10.1111/acel.12495 27240539 PMC5013024

[B74] Wei G. Kibler K. K. Koehler R. C. Maruyama T. Narumiya S. Doré S. (2008). Prostacyclin receptor deletion aggravates hippocampal neuronal loss after bilateral common carotid artery occlusion in mouse. Neuroscience 156, 1111–1117. 10.1016/j.neuroscience.2008.07.073 18790018 PMC6010173

[B75] Womack T. R. Vollert C. Nwoko O. Schmitt M. Montazari S. Beckett T.. (2020). Prostacyclin promotes degenerative pathology in a model of Alzheimer’s disease. bioRxiv [Preprint]. 10.1101/2020.04.15.039842 PMC8860182 35197825

[B76] Yang C. DeMars K. M. Alexander J. C. Febo M. Candelario-Jalil E. (2017). Sustained neurological recovery after stroke in aged rats treated with a novel prostacyclin analog. Stroke 48, 1948–1956. 10.1161/STROKEAHA.117.016474 28588054 PMC5508605

[B77] Yermakova A. V. Rollins J. Callahan L. M. Rogers J. O’Banion M. K. (1999). Cyclooxygenase-1 in human Alzheimer and control brain: quantitative analysis of expression by microglia and CA3 hippocampal neurons. J. Neuropathol. Exp. Neurol. 58, 1135–1146. 10.1097/00005072-199911000-00003 10560656

[B78] Zhen G. Kim Y. T. Li R. Yocum J. Kapoor N. Langer J.. (2012). PGE2 EP1 receptor exacerbated neurotoxicity in a mouse model of cerebral ischemia and Alzheimer’s disease. Neurobiol. Aging 33, 2215–2219. 10.1016/j.neurobiolaging.2011.09.017 22015313 PMC3299840

[B79] Zlokovic B. V. (2011). Neurovascular pathways to neurodegeneration in Alzheimer’s disease and other disorders. Nat. Rev. Neurosci. 12, 723–738. 10.1038/nrn3114 22048062 PMC4036520

